# Daily digital gaming, weekly training duration, and perceived fatigue in athletes: indirect statistical associations involving digital game addiction awareness

**DOI:** 10.3389/fpsyt.2026.1945442

**Published:** 2026-10-30

**Authors:** Okan Kılıçkaya, Yunus Şahinler, Buşra Özcan, Çetin Tan, Önder Şanlı, Melda Sunay, Sabri Can Metin, İsmail Belli, Faik Öz, Levent Ceylan

**Affiliations:** 1 Department of Software Engineering, Faculty of Engineering and Architecture, Istanbul Gelişim University, Istanbul, Türkiye; 2 Faculty of Sport Sciences, Istanbul Gelisim University, Istanbul, Türkiye; 3 School of Physical Education and Sports, Siirt University, Siirt, Türkiye; 4 Department of Physical Education and Sports Teaching, Faculty of Sports Sciences, Firat University, Elazig, Türkiye; 5 Department of Knowledge Management, Besni Ali Erdemoğlu Vocational School, Adıyaman University, Adıyaman, Türkiye; 6 Department of Sociology, Faculty of Humanities and Social Sciences, Bursa Technical University, Bursa, Türkiye; 7 Department of Coaching Education, Faculty of Sport Sciences, Istanbul Topkapı University, Istanbul, Türkiye; 8 Department of Exercise and Sport Sciences, Faculty of Sport Sciences, Haliç University, Istanbul, Türkiye; 9 Red and River Digital Publishing and Trade Limited Company, Rize, Türkiye; 10 Department of Coaching Education, Faculty of Sport Sciences, Hitit University, Çorum, Türkiye

**Keywords:** athletes, cognitive fatigue, digital gaming, gaming addiction awareness, physical fatigue, structural equation modeling

## Abstract

**Introduction:**

Digital gaming is a common leisure activity among athletes, yet its associations with weekly training duration and perceived fatigue remain insufficiently understood. This study examined the direct and indirect associations among daily digital gaming duration, weekly training duration, digital game addiction awareness, and perceived physical and cognitive fatigue.

**Methods:**

A cross-sectional correlational design was employed with 587 adult athletes from team and individual sports in Istanbul, Türkiye. Data were collected using the Awareness of Digital Game Addiction Scale and the Scale of Physical and Cognitive Fatigue Perceived. Structural equation modeling with bias-corrected bootstrapping was performed.

**Results:**

Daily digital gaming duration (β = .41, p < .001) and weekly training duration (β = .24, p < .001) were positively associated with digital game addiction awareness, which was associated with perceived physical fatigue (β = .32, p < .001) and cognitive fatigue (β = .38, p < .001). Significant indirect associations involving digital game addiction awareness were observed for both gaming and training duration within the specified model. The model showed acceptable fit (χ^2^(4) = 9.64, CFI = .95, RMSEA = .049, SRMR = .041).

**Discussion:**

The findings indicate significant concurrent associations among the study variables. However, given the cross-sectional design, the indirect associations should not be interpreted as evidence of temporal or causal mediation.

## 1 Introduction

Digital gaming has become an increasingly common form of leisure and digital engagement across different age and social groups. Its accessibility through smartphones, computers, and online platforms has contributed to longer and more frequent gaming sessions, while also increasing research interest in the circumstances under which gaming engagement may become problematic (1). Athletes may represent a particularly relevant population in this context because digital gaming habits coexist with training demands, recovery requirements, and the cognitive demands associated with sport participation (2). Prolonged gaming engagement may be associated with athletes’ perceived physical and cognitive fatigue, particularly when gaming time overlaps with or competes with sleep, recovery, or other restorative activities.

Problematic gaming is characterized by impaired control over gaming, increased priority given to gaming over other activities, and continued engagement despite negative consequences. The recognition of gaming disorder within the International Classification of Diseases has strengthened concerns regarding the psychological, behavioral, and functional consequences of excessive gaming (3). Previous evidence indicates that problematic gaming may be associated with maladaptive coping, psychological symptoms, reduced social functioning, and poorer daily functioning (4). Such consequences may be particularly relevant for athletes, whose daily routines require effective time management, adequate recovery, sustained attention, and regular training participation. Excessive digital gaming has been associated with fatigue, sleep disturbances, depressive and anxiety-related symptoms, and social isolation (5). Late-night gaming may increase cognitive arousal and screen exposure, which can delay sleep onset, reduce sleep duration, and contribute to daytime fatigue and impaired daily functioning (6, 7). These associations indicate that digital gaming should be considered not only as a leisure behavior but also as a potential factor related to psychological and physical functioning. Importantly, longer gaming duration should not be interpreted as evidence of problematic gaming or gaming disorder, as the present study did not assess impaired control, functional impairment, prioritization of gaming, or other diagnostic symptoms. Accordingly, evidence concerning problematic gaming and its consequences should be regarded as contextual background rather than as direct support for the role of digital game addiction awareness in the present structural model.

Athletes may experience high levels of mental fatigue in association with competition stress and intense training loads, with potential implications for endurance performance, motor skills performance, and decision-making performance (8). Perceived fatigue may reduce athletes’ physical and cognitive capacity, while digital gaming, particularly when prolonged or occurring at times that may interfere with recovery, has been associated with sleep disturbances and less favorable recovery-related experiences (9).

Evidence from sport settings suggests that digital media use immediately before performance may be associated with poorer sport-specific cognitive functioning. Similarly, research conducted among volleyball players has examined associations between digital gaming and sport-related physical and performance characteristics, including shoulder flexibility, muscle strength, and reaction speed (10). Smartphone and video game use before competition has been associated with poorer passing accuracy and decision-making among soccer players (11), while similar exposure has been linked to reduced attacking and defensive decision-making in amateur boxers. More broadly, mental fatigue has been associated with declines in physical performance, technical execution, and decision-making across athletic contexts (12), providing additional context for examining perceived fatigue alongside digital engagement in athletes.

Digital game addiction awareness refers to individuals’ awareness of the characteristics and potential adverse consequences associated with digital game addiction. Importantly, this construct reflects individuals’ knowledge and beliefs regarding gaming-related harms and should not be interpreted as an indicator of problematic gaming behavior, functional impairment, diagnostic symptoms, or addiction severity. The construct comprises internal awareness and external awareness dimensions, reflecting awareness arising from individuals’ perceptions of their internal experiences and external environment, respectively. Higher scores indicate greater awareness of digital game addiction and should not be interpreted as indicating a higher level or greater severity of digital game addiction. Thus, the construct assessed in the present study concerns awareness of gaming-related risks and consequences rather than the presence or severity of problematic gaming itself. Some empirical evidence suggests that gaming exposure may be associated with gaming-related awareness, although the direction of this relationship is not consistent across studies (13–15). However, a specific theoretical or empirical basis for expecting greater digital game addiction awareness to be associated with greater perceived fatigue has not been established. Accordingly, the awareness–fatigue association was examined exploratorily rather than treated as an established psychological pathway.

Weekly training duration was included in the model as a sport-related contextual indicator rather than as a direct marker of psychological or behavioral dysfunction. Training exposure is closely intertwined with athletes’ recovery demands, fatigue status, perceived well-being, and the broader monitoring of daily training responses (16–18). Contemporary athlete-monitoring frameworks further emphasize that training load should be interpreted within a multidimensional context that includes recovery and subjective well-being rather than as an isolated exposure variable (19, 20). Accordingly, weekly training duration was retained as a contextual variable representing the broader training–recovery environment of the athletes. Because a specific theoretical link between training duration and digital game addiction awareness has not been established, this association was examined exploratorily within the structural model rather than treated as a theoretically established pathway.

Despite growing interest in problematic gaming, athlete recovery, and mental fatigue, evidence examining how daily digital gaming duration, weekly training duration, digital game addiction awareness, and perceived physical and cognitive fatigue coexist within the same athlete sample remains limited. Existing literature provides stronger support for several pairwise associations among digital engagement, training demands, recovery, and fatigue than for the complete structural sequence examined in the present study. Accordingly, the present study aimed to examine the direct and indirect statistical associations among these variables within a cross-sectional structural model. Digital game addiction awareness was treated as an awareness-related construct rather than as an indicator of problematic gaming or addiction severity, and the proposed indirect pathways were interpreted as concurrent statistical associations rather than as evidence of a causal mechanism. Thus, the ordering of variables was used as an exploratory analytical specification rather than as a representation of an established temporal or psychological process. This analytical ordering was selected to examine the concurrent statistical associations of digital game addiction awareness with both the exposure variables and perceived fatigue outcomes within the specified model, rather than to imply that awareness functions as a temporally or theoretically established mediator.

RQ1: Are daily digital gaming duration and weekly training duration associated with athletes’ digital game addiction awareness?
RQ2: Is digital game addiction awareness associated with athletes’ perceived physical and cognitive fatigue?
RQ3: Do daily digital gaming duration and weekly training duration show indirect statistical associations with perceived physical and cognitive fatigue involving digital game addiction awareness within the proposed cross-sectional structural model?

## 2 Method

### 2.1 Research model

This study employed a quantitative, cross-sectional, and correlational design to examine the direct and indirect associations among daily digital gaming duration, weekly training duration, digital game addiction awareness, and perceived physical and cognitive fatigue in athletes. Daily digital gaming duration and weekly training duration were specified as exogenous variables, digital game addiction awareness was specified as an intermediate awareness-related construct for estimating indirect statistical associations, and perceived physical fatigue and perceived cognitive fatigue were specified as outcome variables. The proposed model examined the associations of daily digital gaming duration and weekly training duration with digital game addiction awareness, the associations of digital game addiction awareness with perceived physical and cognitive fatigue, and the indirect associations between the exogenous variables and the outcome variables through digital game addiction awareness. Given the cross-sectional design, the estimated indirect effects were interpreted as statistical associations rather than evidence of a temporal or causal process. In this context, an indirect statistical association refers to the product of two concurrent estimated associations: the association between an exogenous variable and digital game addiction awareness and the association between digital game addiction awareness and a fatigue outcome. It therefore describes a pattern within the specified covariance structure rather than a process occurring sequentially over time. The ordering of variables in the model was analytical rather than temporal or causal. Because all variables were measured concurrently, the estimated indirect effects should not be interpreted as evidence that gaming or training duration increases awareness or that awareness subsequently causes fatigue.

### 2.2 Population and sample

The study group consisted of 587 athletes recruited through convenience sampling from sports clubs operating in the Ataşehir district of Istanbul, Türkiye. Participants were involved in team sports, such as football, basketball, and volleyball, or individual sports, such as tennis, athletics, and swimming. Eligible participants were required to be at least 18 years old, actively participating in regular sports training, affiliated with a sports club at the time of data collection, and willing to participate voluntarily. Athletes who did not complete all data collection instruments were excluded from the analyses. The final analytical sample consisted of 587 athletes with complete data. Accordingly, all analyses were conducted using complete cases, and no missing values remained in the final analytical sample. Given the convenience sampling procedure and the geographically restricted recruitment setting, the sample should not be considered representative of all athletes in Istanbul or Türkiye.

### 2.3 Data collection tools

Data were collected using a Personal Information Form, the Awareness of Digital Game Addiction Scale (ADGAS), and the Scale of Physical and Cognitive Fatigue Perceived (SPCFP). The Personal Information Form was used to obtain participants’ demographic and sport-related characteristics, including age, gender, education level, sport type, self-reported weekly training duration, and self-reported daily digital gaming duration. The ADGAS was used to assess participants’ awareness of digital game addiction, whereas the SPCFP was used to assess their perceived physical and cognitive fatigue.

#### 2.3.1 Personal information form

The Personal Information Form was developed by the research team to collect participants’ demographic and sport-related information. The form included items on age, gender, education level, sport type, self-reported weekly training duration, and self-reported daily digital gaming duration.

#### 2.3.2 Awareness of digital game addiction scale

The Awareness of Digital Game Addiction Scale (ADGAS; original Turkish name: *Dijital Oyun Bağımlılığına İlişkin Farkındalık Ölçeği* [DOBİFÖ]) was developed by Tekkurşun Demir and Cicioğlu (21) to assess individuals’ awareness of digital game addiction. The scale consists of 12 items and two subdimensions: Internal Awareness and External Awareness. Internal Awareness comprises five items and reflects awareness of the internal and emotional consequences associated with digital game addiction, whereas External Awareness comprises seven items and reflects awareness of its potential consequences for social, school, work, and other environmental domains. Thus, the scale assesses individuals’ knowledge, understanding, and awareness of the potential internal and external consequences of digital game addiction rather than the severity of addictive gaming behavior itself. The ADGAS is rated on a five-point Likert-type scale ranging from 1 (*strongly disagree*) to 5 (*strongly agree*). Item 11 is negatively worded and is reverse-scored. Total scores range from 12 to 60, with higher scores indicating greater awareness of digital game addiction. Importantly, higher scores should not be interpreted as indicating greater digital game addiction severity or problematic gaming behavior. In the original validation study, the Cronbach’s alpha coefficients were.82 for Internal Awareness,.83 for External Awareness, and.88 for the total scale.

#### 2.3.3 Scale of physical and cognitive fatigue perceived

The Scale of Physical and Cognitive Fatigue Perceived (SPCFP; original Turkish name: *Algılanan Fiziksel ve Bilişsel Yorgunluk Ölçeği* [AFBYÖ]) was developed by Tekkurşun Demir et al. (22) to assess athletes perceived physical and cognitive fatigue. The 19-item scale consists of two subdimensions: Perceived Physical Fatigue (8 items) and Perceived Cognitive Fatigue (11 items). Participants respond to the items based on how they have felt during the preceding days. The scale is rated on a five-point Likert-type scale ranging from 1 (*strongly disagree*) to 5 (*strongly agree*). Higher scores indicate greater perceived fatigue, with total scores ranging from 19 to 95. In the original validation study, Cronbach’s alpha coefficients were.95 for Perceived Physical Fatigue,.97 for Perceived Cognitive Fatigue, and.97 for the total scale, and the two-factor structure was supported by confirmatory factor analysis.

### 2.4 Ethics committee approval

This study was approved by the Siirt University Ethics Committee (Decision Date: October 3, 2025; Session No: 1288). Participation was voluntary, and informed consent was obtained from all participants before data collection. Participants were informed about the purpose of the study, the confidentiality of their responses, and their right to withdraw from the study at any time without penalty. No personally identifiable information was collected, and all data were handled anonymously in accordance with established research ethics principles.

### 2.5 Analysis of data

IBM SPSS Statistics version 26.0 was used to conduct descriptive statistics, internal consistency analyses, univariate normality assessments, and Pearson correlation analyses. Frequencies and percentages were calculated to summarize participants’ demographic and sport-related characteristics. Means, standard deviations, minimum and maximum values, skewness, and kurtosis coefficients were calculated for the study variables. Skewness and kurtosis values within ±1 were considered indicative of approximately normal distributions. Cronbach’s alpha coefficients were calculated to evaluate the internal consistency of the scales and their subdimensions. Daily digital gaming duration and weekly training duration were collected as open-ended, self-reported time variables expressed in hours. These variables were categorized only for the descriptive presentation of participant characteristics in **Table 1**. The original continuous values were used in the structural model analyses. Pearson correlation analysis was conducted to examine the direction and magnitude of the associations among digital game addiction awareness, perceived physical fatigue, and perceived cognitive fatigue.

**Table 1:** Demographic and sport-related characteristics of the participants (N = 587).

Variable	Category	N	%
Gender	Female	256	43.6
	Male	314	53.5
	Prefer not to say	17	2.9
Age Group	18–20 years	148	25.2
	21–23 years	182	31.0
	24–26 years	156	26.6
	27 years and above	101	17.2
Education Level	High School	134	22.8
	Associate Degree	119	20.3
	Bachelor’s Degree	262	44.6
	Graduate Degree	72	12.3
Sport Type	Team Sports	329	56.0
	Individual Sports	231	39.4
	Both team and individual sports	27	4.6
Weekly Training Duration	1–3 hours	94	16.0
	4–6 hours	183	31.2
	7–9 hours	176	30.0
	10 hours or more	134	22.8
Daily Digital Gaming Duration	No digital gaming	121	20.6
	Less than 1 hour	144	24.5
	1–2 hours	162	27.6
	3–4 hours	103	17.5
	5 hours or more	57	9.8

Weekly training duration and daily digital gaming duration were collected as open-ended, self-reported variables expressed in hours. The variables were categorized only for descriptive presentation in **Table 1**, whereas their original continuous values were used in the structural model analyses.

Structural equation modeling was performed using maximum likelihood estimation. Prior to model estimation, univariate and multivariate distributional characteristics and potential multivariate outliers were examined. No severe violations requiring transformation or case exclusion were identified. Visual inspection of scatterplots indicated no substantial departures from linearity among the focal continuous variables. Multivariate outliers were assessed using Mahalanobis distance, and no observations were excluded from the final analyses. No *post-hoc* correlated error terms or data-driven model modifications were introduced into the structural model.

IBM SPSS AMOS version 24.0 was used to test the proposed structural model. Before testing the structural model, confirmatory factor analyses (CFA) were conducted to evaluate the measurement structure of the Awareness of Digital Game Addiction Scale (ADGAS) and the Scale of Physical and Cognitive Fatigue Perceived (SPCFP). The hypothesized two-factor structure of each instrument was tested using item-level indicators. Model fit was evaluated using χ²/df, CFI, TLI, RMSEA, and SRMR. Standardized factor loadings, composite reliability (CR), and average variance extracted (AVE) were also examined to evaluate the reliability and convergent validity of the measurement model. Daily digital gaming duration and weekly training duration were specified as exogenous variables, digital game addiction awareness was specified as an intermediate awareness-related construct for estimating indirect statistical associations, and perceived physical fatigue and perceived cognitive fatigue were specified as outcome variables. The supplementary total fatigue score was not included in the structural model; perceived physical fatigue and perceived cognitive fatigue were retained as separate outcome variables. In the focal structural model, covariance parameters were not specified between daily digital gaming duration and weekly training duration or between the residuals of perceived physical and cognitive fatigue. The focal model was restricted to the structural associations specified in the proposed analytical framework and was not intended to represent all possible covariance relationships among the study variables. Accordingly, the absence of these covariance parameters should be regarded as a model-specification constraint rather than as evidence that the corresponding variables or residual components are empirically independent. In the structural model, the total digital game addiction awareness score was used as an observed composite variable. Model fit was evaluated using the chi-square to degrees of freedom ratio (χ²/df), Comparative Fit Index (CFI), Root Mean Square Error of Approximation (RMSEA), and Standardized Root Mean Square Residual (SRMR). Values below 3 for χ²/df, values of.90 or higher for CFI, and values of.08 or lower for RMSEA and SRMR were considered indicative of acceptable model fit. Direct and indirect effects were estimated simultaneously within the proposed structural model. The direct paths included in the focal structural model were limited to the associations specified within the proposed analytical framework. Other possible direct associations between the exogenous variables and the fatigue outcomes were not assumed to be theoretically absent; rather, they were outside the focal specification of the present model. Accordingly, the reported structural model should be regarded as a parsimonious representation of the focal analytical relationships rather than as an exhaustive model of all possible direct associations among the study variables. The statistical significance of the indirect effects was evaluated using bias-corrected bootstrapping with 5,000 resamples and 95% confidence intervals. An indirect effect was considered statistically significant when its confidence interval did not include zero. Given the cross-sectional design, these indirect effects were interpreted substantively as indirect statistical associations within the specified model rather than as evidence of mediation, temporal sequencing, or a causal process. The ordering of variables in the model was analytical rather than temporal or causal; therefore, the estimated indirect effects should not be interpreted as evidence that gaming or training duration increases awareness or that awareness subsequently causes fatigue. The statistical significance level was set at p <.05.

## 3 Results

Following the presentation of participants’ demographic and sport-related characteristics, the distributions of the scale scores were examined using histograms with superimposed normal curves. The descriptive statistics for daily digital gaming duration and weekly training duration are presented in **Table 2**. The distributions of total digital game addiction awareness, Internal Awareness, External Awareness, total perceived physical and cognitive fatigue, Perceived Physical Fatigue, and Perceived Cognitive Fatigue are presented in **Figure 1**.

**Figure 1:**
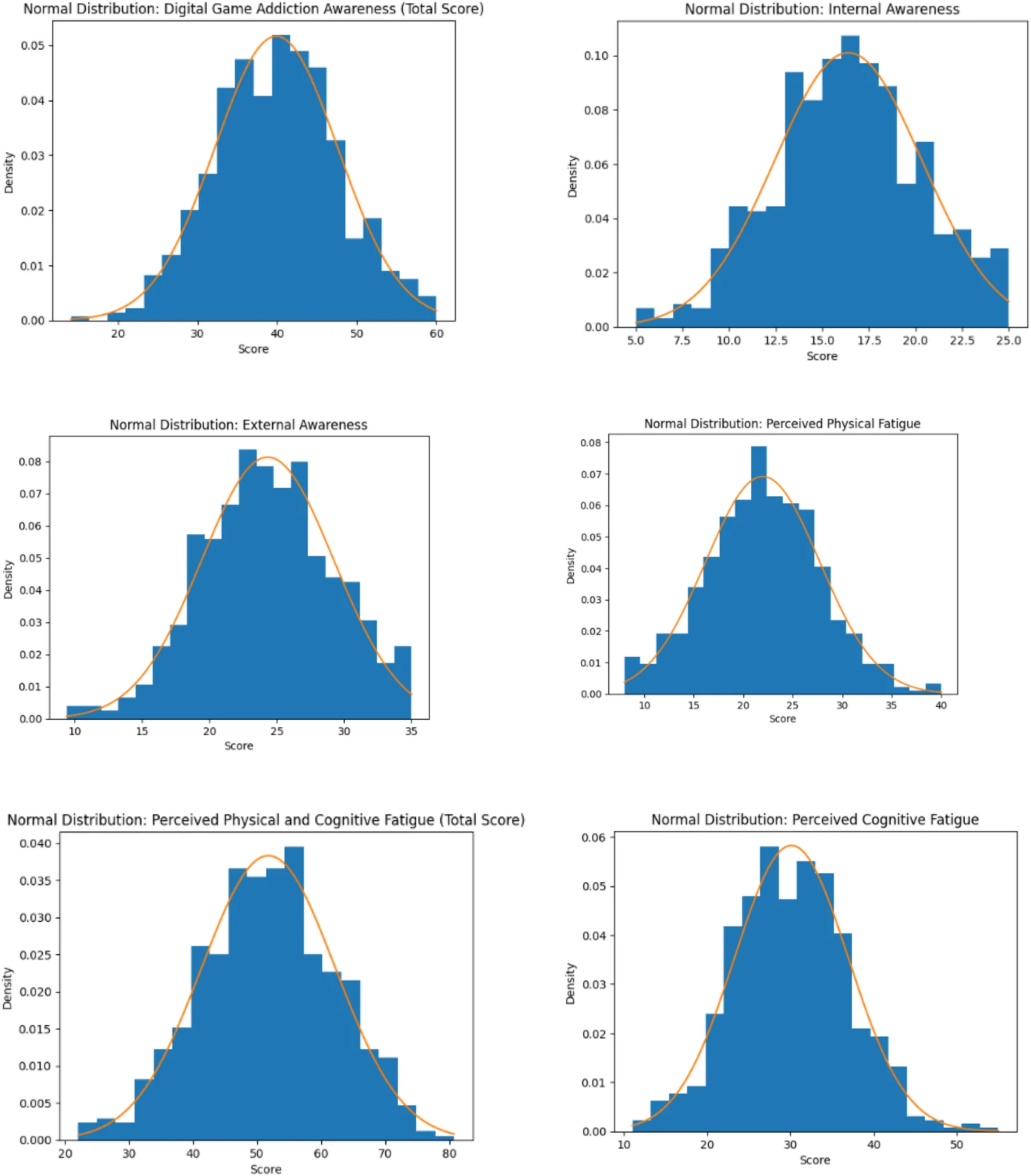
Histograms with superimposed normal curves for the study variables.

**Table 2:** Descriptive statistics for daily digital gaming duration and weekly training duration.

Variable	Min	Max	Mean	SD	Skewness	Kurtosis
Daily Digital Gaming Duration (h/day)	0.00	8.00	1.74	1.63	0.86	0.42
Weekly Training Duration (h/week)	1.00	18.00	7.21	3.46	0.53	−0.18

Daily digital gaming duration is expressed in hours per day, and weekly training duration in hours per week. SD, standard deviation. Skewness and kurtosis values were used to assess the distributional characteristics of the continuous variables.

**Figure 1** presents the histogram plots with superimposed normal distribution curves for the study variables, including Digital Game Addiction Awareness (Total Score), Internal Awareness, External Awareness, Perceived Physical and Cognitive Fatigue (Total Score), Perceived Physical Fatigue, and Perceived Cognitive Fatigue. Visual inspection indicates that all scale-score distributions are approximately bell-shaped and symmetric around their means.

**Table 3** presents the confirmatory factor analysis, composite reliability, and average variance extracted results for the measurement constructs. Standardized factor loadings ranged from.68 to.81 across the four dimensions. Standardized residual variances ranged from.34 to.54 across the measurement indicators, indicating acceptable levels of unexplained variance. Composite reliability values ranged from.875 to.937, indicating satisfactory construct reliability. In addition, AVE values ranged from.558 to.585, supporting adequate convergent validity. The two-factor measurement model of the Awareness of Digital Game Addiction Scale (ADGAS) demonstrated acceptable fit, χ²(53) = 116.20, χ²/df = 2.19, CFI = .965, TLI = .956, RMSEA = .045, 90% CI [.034,.056], and SRMR = .037. These findings support the adequacy of the proposed Internal Awareness and External Awareness factor structure in the present sample. The two-factor measurement model of the Scale of Physical and Cognitive Fatigue Perceived (SPCFP) also demonstrated acceptable fit, χ²(151) = 342.70, χ²/df = 2.27, CFI = .958, TLI = .951, RMSEA = .046, 90% CI [.040,.052], and SRMR = .039. These findings support the adequacy of the proposed Perceived Physical Fatigue and Perceived Cognitive Fatigue factor structure in the present sample.

**Table 3:** Confirmatory factor analysis, composite reliability, and convergent validity results.

Construct	Items	Standardized factor loadings	Standardized Residual variance	CR	AVE
Internal Awareness	5	.72–.81	.34–.48	.875	.585
External Awareness	7	.68–.80	.36–.54	.898	.558
Perceived Physical Fatigue	8	.70–.81	.34–.51	.914	.571
Perceived Cognitive Fatigue	11	.69–.81	.34–.52	.937	.576

**Table 4** presents the descriptive statistics, reliability coefficients, and normality results of the study variables. The Cronbach’s alpha coefficients ranged between.84 and.91, indicating that the measurement instruments demonstrated high internal consistency. The skewness and kurtosis values for all variables were within the acceptable range (± 1), suggesting that the data were approximately normally distributed. Therefore, the assumptions required for parametric statistical analyses were satisfied.

**Table 4:** Descriptive statistics, reliability, and normality results of the study variables (N = 587).

Variables	Min	Max	Mean	SD	Cronbach’s α	Skewness	Kurtosis
Digital Game Addiction Awareness (Total Score)	18	60	40.71	7.73	.88	0.05	-0.09
Internal Awareness	6	25	16.39	3.95	.84	-0.09	-0.19
External Awareness	10	35	24.32	4.91	.86	-0.08	-0.17
Perceived Physical and Cognitive Fatigue (Total Score)	24	82	52.08	10.42	.91	-0.09	-0.26
Perceived Physical Fatigue	9	40	21.96	5.77	.87	0.04	-0.00
Perceived Cognitive Fatigue	12	55	30.12	6.85	.89	0.12	0.09

N = 587. Cronbach’s alpha values above.70 indicate acceptable internal consistency. Skewness and kurtosis values within the range of ±1 indicate that the data meet the assumption of normal distribution.

**Table 5:** Correlation matrix of the study variables (N = 587).

Variables	1	2	3	4	5	6
1. Digital Game Addiction Awareness (Total Score)	–					
2. Internal Awareness	.83**	–				
3. External Awareness	.89**	.48**	–			
4. Perceived Physical Fatigue	.29**	.24**	.26**	–		
5. Perceived Cognitive Fatigue	.34**	.28**	.31**	.52**	–	
6. Perceived Physical and Cognitive Fatigue (Total Score)	.37**	.30**	.33**	.85**	.89**	–

*p <.01.*

According to **Table 5**, all correlations between the variables are positive and significant at the p <.01 level.

There is a low-level positive relationship between the total score for digital game addiction awareness and perceived physical fatigue (r = .29). The relationship between the total awareness score and cognitive fatigue is also positive and falls within the low-to-moderate range (r = .34). The relationship between digital game addiction awareness and total fatigue is r = .37, indicating a low-to-moderate positive association. Accordingly, as awareness scores regarding digital game addiction increase, the physical and cognitive fatigue scores reported by the athletes also tend to rise. However, since correlation analysis does not demonstrate causality, it should not be interpreted to mean that awareness causes fatigue. The relationships between internal awareness and physical fatigue (r = .24) and cognitive fatigue (r = .28) are at a low level. The relationship between external awareness and physical fatigue is r = .26, while the relationship with cognitive fatigue is r = .31. These values ​​reveal that external awareness shows a relatively stronger relationship-particularly with cognitive fatigue-compared to internal awareness. The relationship between internal and external awareness (r = .48) indicates that the two sub-dimensions are moderately related but do not represent the exact same construct. The correlation between physical and cognitive fatigue (r = .52) also indicates that the two dimensions are moderately related while representing distinct aspects of perceived fatigue.

**Table 6** presents the bias-corrected bootstrapped indirect effects estimated within the structural equation model. Daily digital game playing time showed a statistically significant indirect association with perceived physical fatigue through digital game addiction awareness (B = .15, BootSE = .04, β = .13, 95% BC CI [.07,.23]). A slightly stronger indirect association was observed between daily digital game playing time and perceived cognitive fatigue through digital game addiction awareness (B = .17, BootSE = .05, β = .16, 95% BC CI [.09,.26]). Weekly training duration also showed a statistically significant indirect association with perceived physical fatigue through digital game addiction awareness (B = .22, BootSE = .06, β = .08, 95% BC CI [.10,.34]) and with perceived cognitive fatigue (B = .10, BootSE = .03, β = .09, 95% BC CI [.04,.17]). Since none of the bias-corrected confidence intervals included zero, all four indirect effects were considered statistically significant. Based on the standardized coefficients, the strongest indirect association was observed between daily digital game playing time and perceived cognitive fatigue (β = .16), whereas the weakest indirect association was observed between weekly training duration and perceived physical fatigue (β = .08). Given the cross-sectional design of the study, these findings should be interpreted as statistically significant indirect associations rather than evidence of causal mediation. These estimates reflect concurrent statistical relationships within the specified model; they do not establish temporal precedence or a sequential process among gaming or training duration, awareness, and fatigue, and alternative directional or third-variable explanations cannot be excluded. The corresponding total standardized effects were.30 for gaming duration on perceived physical fatigue,.16 for gaming duration on perceived cognitive fatigue,.08 for weekly training duration on perceived physical fatigue, and.29 for weekly training duration on perceived cognitive fatigue.

**Table 6:** Bias-corrected bootstrapped indirect effects in the structural equation model.

Indirect path	B	BootSE	β	95% BC CI	Direct β	Total β
Gaming → Awareness → Physical Fatigue	.15	.04	.13	[.07,.23]	.17	.30
Gaming → Awareness → Cognitive Fatigue	.17	.05	.16	[.09,.26]	-	.16
Training → Awareness → Physical Fatigue	.22	.06	.08	[.10,.34]	-	.08
Training → Awareness → Cognitive Fatigue	.10	.03	.09	[.04,.17]	.20	.29

B, unstandardized indirect effect; BootSE, bootstrap standard error; β, standardized effect; BC CI , bias-corrected confidence interval. Total standardized effects were calculated as the sum of the corresponding direct and indirect standardized effects. A dash (-) indicates that no direct path was specified in the focal structural model.

A sensitivity analysis was conducted by adjusting the structural model for age, gender, education level, and sport type. The focal structural associations remained statistically significant and showed only minor changes in magnitude after adjustment. The four indirect associations also remained statistically significant, as none of the bias-corrected 95% confidence intervals included zero. Overall, adjustment for the selected demographic and sport-related covariates did not materially change the pattern of the main findings (**Table 7**).

**Table 7:** Sensitivity analysis of the structural model adjusted for demographic and sport-related covariates.

Structural association	Original β	Adjusted β	p/95% BC CI
Daily Digital Gaming Duration → Digital Game Addiction Awareness	.41	.39	<.001
Weekly Training Duration → Digital Game Addiction Awareness	.24	.22	<.001
Digital Game Addiction Awareness → Perceived Physical Fatigue	.32	.31	<.001
Digital Game Addiction Awareness → Perceived Cognitive Fatigue	.38	.36	<.001
Daily Digital Gaming Duration → Perceived Physical Fatigue	.17	.15	.002
Weekly Training Duration → Perceived Cognitive Fatigue	.20	.18	<.001
Gaming → Awareness → Physical Fatigue	.13	.12	[.07,.18]
Gaming → Awareness → Cognitive Fatigue	.16	.14	[.09,.20]
Training → Awareness → Physical Fatigue	.08	.07	[.03,.12]
Training → Awareness → Cognitive Fatigue	.09	.08	[.04,.13]

The adjusted model included age, gender, education level, and sport type as covariates. β, standardized coefficient; BC CI, bias-corrected confidence interval. Indirect effects were evaluated using 5,000 bootstrap resamples. Confidence intervals that did not include zero were considered statistically significant.

A sensitivity analysis was conducted after excluding the 121 participants who reported no digital gaming. In the gamers-only subsample (N = 466), the direction and magnitude of the focal structural associations were highly similar to those observed in the full sample. Daily digital gaming duration and weekly training duration remained significantly associated with digital game addiction awareness, and digital game addiction awareness remained significantly associated with both perceived physical and cognitive fatigue. The two focal direct associations also remained statistically significant. Furthermore, all four indirect associations remained statistically significant because their bias-corrected 95% confidence intervals did not include zero. Overall, restricting the analysis to participants who reported digital gaming did not materially change the pattern of the main findings.

**Figure 2** presents the structural equation model examining the relationships between daily digital game playing time, weekly training duration, digital game addiction awareness, and perceived fatigue among athletes. The results indicated that daily digital game playing time was significantly associated with digital game addiction awareness (β = .41, p <.001). Similarly, weekly training duration was significantly and positively associated with digital game addiction awareness (β = .24, p <.001). These findings indicate that athletes who reported longer digital gaming durations and higher weekly training durations also tended to report greater awareness of digital game addiction. Furthermore, digital game addiction awareness was significantly associated with both perceived physical fatigue (β = .32, p <.001) and perceived cognitive fatigue (β = .38, p <.001). These results indicate that higher levels of digital game addiction awareness were concurrently associated with higher levels of physical and cognitive fatigue among athletes (**Table 8**). The model also included statistically significant direct associations. Specifically, daily digital game playing time was directly associated with perceived physical fatigue (β = .17, p = .002), while weekly training duration was directly associated with perceived cognitive fatigue (β = .20, p <.001). Given the cross-sectional design, these paths represent statistical associations and should not be interpreted as evidence of temporal or causal effects **Table 9**.

**Figure 2:**
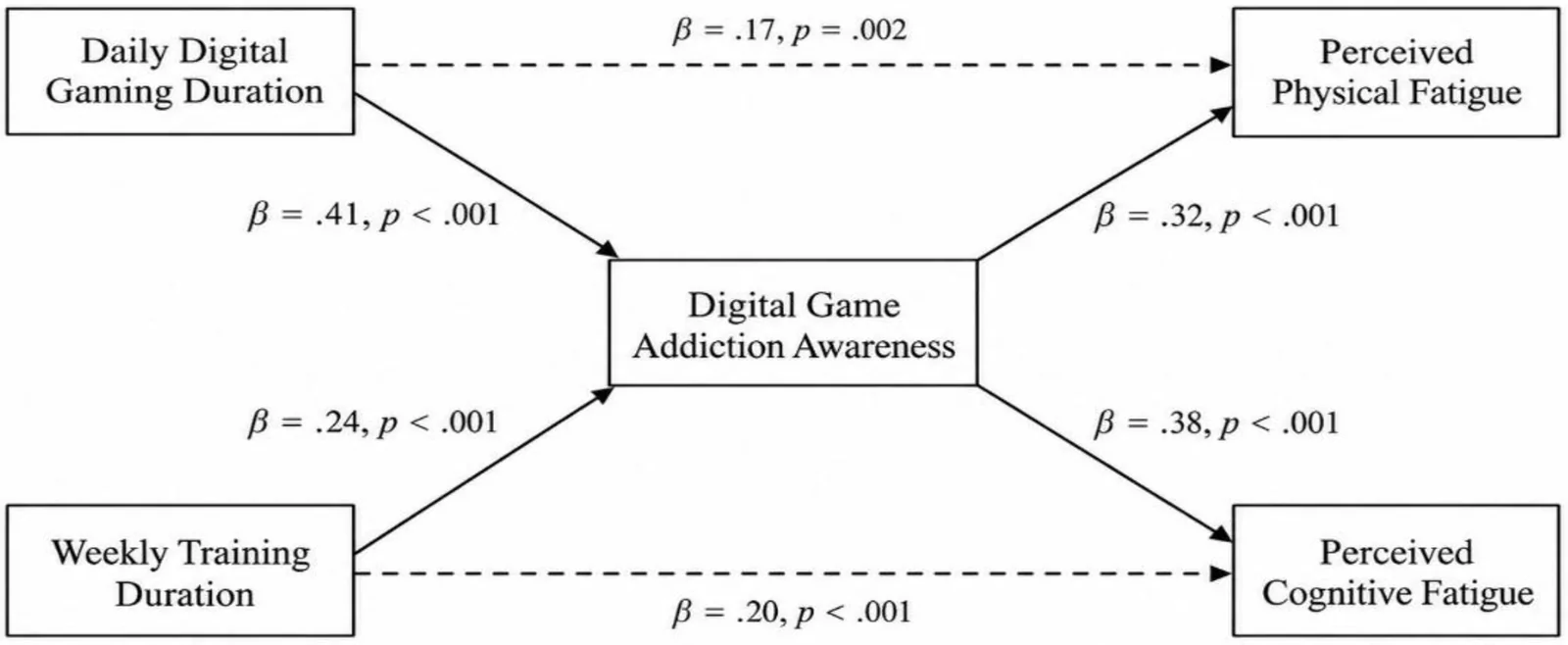
Structural model of digital gaming, training, awareness, and perceived fatigue. Standardized path coefficients (β) are presented. Solid arrows represent the specified structural paths, whereas dashed arrows represent additional direct associations included in the model. **p <.01, ***p <.001.

**Table 8:** Sensitivity analysis restricted to participants reporting digital gaming (N = 466).

Structural association	Full sample β	Gamers-Only β	p/95% BC CI
Daily Digital Gaming Duration → Digital Game Addiction Awareness	.41	.39	<.001
Weekly Training Duration → Digital Game Addiction Awareness	.24	.23	<.001
Digital Game Addiction Awareness → Perceived Physical Fatigue	.32	.31	<.001
Digital Game Addiction Awareness → Perceived Cognitive Fatigue	.38	.37	<.001
Daily Digital Gaming Duration → Perceived Physical Fatigue	.17	.16	.002
Weekly Training Duration → Perceived Cognitive Fatigue	.20	.19	<.001
Gaming → Awareness → Physical Fatigue	.13	.12	[.06,.19]
Gaming → Awareness → Cognitive Fatigue	.16	.15	[.08,.22]
Training → Awareness → Physical Fatigue	.08	.07	[.03,.12]
Training → Awareness → Cognitive Fatigue	.09	.08	[.03,.14]

The sensitivity analysis was restricted to participants who reported digital gaming (N = 466). β , standardized coefficient; BC CI , bias-corrected 95% confidence interval. Indirect associations were evaluated using 5,000 bootstrap resamples.

**Table 9:** Model fit indices and explained variance of the structural equation model.

Fit index	Value	Recommended threshold
χ²	9.64	–
df	4	–
p	.047	–
χ²/df	2.41	< 3
CFI	.95	>.90
RMSEA	.049	<.08
RMSEA 90% CI	[.005,.090]	–
SRMR	.041	<.08
R² – Digital Game Addiction Awareness	.23	–
R² – Perceived Physical Fatigue	.18	–
R² – Perceived Cognitive Fatigue	.22	–

Chi-square; df, degrees of freedom; CFI , comparative fit index; RMSEA , root mean square error of approximation; CI , confidence interval; SRMR , standardized root mean square residual; R², explained variance. A dash (-) indicates that no single recommended threshold was applied for the corresponding statistic.

The structural equation model demonstrated an acceptable fit to the data, χ²(4) = 9.64, p = .047, χ²/df = 2.41, CFI = .95, RMSEA = .049, 90% CI [.005,.090], and SRMR = .041. The model explained 23% of the variance in digital game addiction awareness, 18% of the variance in perceived physical fatigue, and 22% of the variance in perceived cognitive fatigue.

## 4 Limitations

This study has several limitations. First, the cross-sectional design does not allow conclusions regarding temporal precedence or causality; therefore, the indirect associations identified in the structural model should not be interpreted as causal mediation. Because all variables were measured concurrently, reverse-direction associations and alternative structural interpretations cannot be ruled out. Second, all main variables were assessed using self-report measures, which may have introduced recall bias and common-method variance. Third, daily digital gaming duration reflects time spent gaming and does not assess problematic gaming behavior, functional impairment, or addiction severity. In addition, potentially relevant factors such as sleep duration and quality, timing of screen use, psychological symptoms, injury status, game genre, caffeine consumption, and other recovery-related behaviors were not assessed and may have influenced the observed associations. Although the gamers-only sensitivity analysis yielded a similar pattern of findings, measurement invariance between gamers and non-gamers was not examined; therefore, potential differences in the interpretation of the ADGAS across these groups cannot be entirely excluded. Finally, the use of convenience sampling from sports clubs in a geographically restricted area limits the generalizability of the findings to broader athlete populations.

## 5 Discussion and conclusion

This study investigated the relationships among athletes’ digital game addiction awareness, perceived fatigue, daily digital gaming duration, and weekly training duration within a structural equation model. With respect to RQ1, daily digital gaming duration and weekly training duration were significantly associated with digital game addiction awareness. Previous studies provide some support for an association between gaming exposure and gaming-related awareness or perceptions. Among athletes and e-sports players, digital game addiction awareness scores have been reported to differ significantly across daily gaming-duration groups, with lower awareness observed among participants reporting longer gaming duration (13). Similarly, gaming-related views among athletes have been reported to vary according to gaming frequency and gaming duration (14), while gaming frequency has also been associated with how university students define and perceive gaming addiction (15). These findings suggest that gaming exposure and addiction-related awareness may be related, although the direction and nature of this association are not yet consistent across studies. By contrast, a specific theoretical basis for the association between weekly training duration and digital game addiction awareness remains limited; therefore, this finding should be interpreted as exploratory and may reflect unmeasured individual or contextual factors rather than an effect of training duration on awareness. With respect to RQ2, digital game addiction awareness was significantly and positively associated with both perceived physical and cognitive fatigue.

With respect to RQ3, significant indirect statistical associations involving digital game addiction awareness were observed between daily digital gaming duration or weekly training duration and perceived physical and cognitive fatigue within the specified cross-sectional structural model. Substantively, these estimates indicate that the observed associations between gaming or training duration and fatigue were statistically represented by the concurrent associations linking these variables with digital game addiction awareness within the specified model. However, these associations should not be interpreted as evidence that gaming or training duration increases awareness or that awareness subsequently causes fatigue. Previous research suggests that mental fatigue may be associated with reduced performance and attentional difficulties in athletes (11, 23), while central nervous system processes related to mental fatigue may influence motivation and cognitive control (24, 25). Previous studies have also reported that cognitively demanding tasks may impair muscle force production and that mental and muscular fatigue may be associated with changes in soccer performance (26, 27). These mechanisms were not directly assessed in the present study and should therefore be considered only as possible contextual explanations for the observed associations. For example, prolonged digital gaming may coexist with greater cognitive stimulation or less favorable recovery-related behaviors, but the present data cannot establish these processes. Alternative explanations should also be considered. Athletes experiencing greater fatigue may be more likely to endorse statements concerning the adverse consequences of digital gaming, while common-method effects arising from concurrent self-report measures may also have contributed to the observed associations. In addition, unmeasured problematic gaming behavior or other third variables may partially account for the relationships identified in the model. Broader evidence from umbrella reviews and meta-analyses has examined factors associated with digital addiction, its prevalence, and its health-related implications (28–30). However, these findings concern digital addiction more broadly and should not be interpreted as direct evidence of addiction severity or causal relationships in the present study. Accordingly, the observed indirect associations should not be regarded as evidence of a temporal or causal process, given the cross-sectional design and the generally small-to-moderate magnitude of the estimated associations. In conclusion, the findings indicate significant concurrent associations among digital gaming engagement, weekly training duration, digital game addiction awareness, and perceived physical and cognitive fatigue in athletes. These findings suggest that digital gaming habits may be relevant to consider alongside other recovery-related behaviors, particularly in athletes with demanding training schedules. Coaches, sport psychologists, and sport scientists may encourage balanced digital engagement without interpreting longer gaming duration or higher awareness scores as evidence of problematic gaming or addiction severity. Future studies should examine these relationships using longitudinal and experimental designs and include objective indicators of sleep, recovery, and sport-specific performance across different sports, age groups, and competitive levels.

## Data Availability

The raw data supporting the conclusions of this article will be made available by the authors, without undue reservation.
